# Informed Consent in Pragmatic Trials: Results from a Survey of Trials published 2014–2019

**DOI:** 10.1136/medethics-2021-107765

**Published:** 2021-11-15

**Authors:** Jennifer Zhe Zhang, Stuart G Nicholls, Kelly Carroll, Hayden P Nix, Cory E Goldstein, Spencer P Hey, Jamie C Brehaut, Paul C McLean, Charles Weijer, Dean A Fergusson, Monica Taljaard

**Affiliations:** School of Epidemiology and Public Health, University of Ottawa, 600 Peter Morand Crescent, Ottawa, Ontario, Canada, K1G 5Z3.; Clinical Epidemiology Program, Ottawa Hospital Research Institute (OHRI), The Ottawa Hospital, Civic Campus, 1053 Carling Avenue, Ottawa, Ontario, Canada, K1Y 4E9.; Clinical Epidemiology Program, Ottawa Hospital Research Institute (OHRI), The Ottawa Hospital, General Campus, 501 Smyth Road, Ottawa, Ontario, Canada, K1H 8L6.; Schulich School of Medicine & Dentistry, Western University, 1151 Richmond St, London, Ontario, Canada, N6A 5C1.; Department of Philosophy, Western University, WIRB 7175, 1151 Richmond Street London, Ontario, Canada, N6A 5B7.; Center for Bioethics, Harvard Medical School, 641 Huntington Avenue, Boston, Massachusetts, United States, 02115.; Clinical Epidemiology Program, Ottawa Hospital Research Institute (OHRI), The Ottawa Hospital; General Campus, 501 Smyth Road, Ottawa, Canada, K1H 8L6; School of Epidemiology and Public Health, University of Ottawa, 600 Peter Morand Crescent, Ottawa, Ontario, Canada, K1G 5Z3.; Unaffiliated, Boston, Massachusetts, United States.; Departments of Medicine, Epidemiology & Biostatistics, and Philosophy, Western University, WIRB 7160, 1151 Richmond Street, London, Ontario, Canada, N6A 5B7.; Clinical Epidemiology Program, Ottawa Hospital Research Institute (OHRI), The Ottawa Hospital, General Campus, 501 Smyth Road, Ottawa, Ontario, Canada, K1H 8L6; Department of Medicine, University of Ottawa, Ottawa, Ontario, Canada; School of Epidemiology and Public Health, University of Ottawa, 600 Peter Morand Crescent, Ottawa, Ontario, Canada, K1G 5Z3.; Clinical Epidemiology Program, Ottawa Hospital Research Institute (OHRI), The Ottawa Hospital, Civic Campus, 1053 Carling Avenue, Ottawa, Ontario, Canada, K1Y 4E9; School of School of Epidemiology and Public Health, University of Ottawa, 600 Peter Morand Crescent, Ottawa, Ontario, Canada, K1G 5Z3.

**Keywords:** Clinical Trial, Ethics- Research, Primary Health Care, Informed Consent, Ethics Committees

## Abstract

**Objectives:**

To describe reporting of informed consent in pragmatic trials, justifications for waivers of consent, and reporting of alternative approaches to standard written consent. To identify factors associated with (a) not reporting, and (b) not obtaining consent.

**Methods:**

Survey of primary trial reports, published 2014–2019, identified using an electronic search filter for pragmatic trials implemented in MEDLINE, and registered in ClinicalTrials.gov.

**Results:**

Among 1988 trials, 132 (6.6%) did not include a statement about participant consent, 1691 (85.0%) reported consent had been obtained, 139 (7.0%) reported a waiver, and 26 (1.3%) reported consent for one aspect (e.g., data collection) but a waiver for another (e.g., intervention). Of the 165 trials reporting a waiver, 76 (46.1%) provided a justification. Few (53, 2.9%) explicitly reported use of alternative approaches to consent. In multivariable logistic regression analyses, lower journal impact factor (p=0.001) and cluster randomisation (p<0.0001) were significantly associated with not reporting on consent, while trial recency, cluster randomisation, higher income country settings, health services research, and explicit labelling as pragmatic were significantly associated with not obtaining consent (all p-values<0.0001).

**Discussion:**

Not obtaining consent seems to be increasing and is associated with the use of cluster randomisation and pragmatic aims, but neither cluster randomisation nor pragmatism are currently accepted justifications for waivers of consent. Rather than considering either standard written informed consent or waivers of consent, researchers and research ethics committees could consider alternative consent approaches that may facilitate the conduct of pragmatic trials while preserving patient autonomy and the public’s trust in research.

## INTRODUCTION

Randomised controlled trials are a robust study design for providing evidence about effectiveness of interventions. However, randomised controlled trials are not homogenous and differ in their purpose, scope, and methodological features. One way in which trials differ is in whether their intention is pragmatic or explanatory. Pragmatic trials are designed with the intent to inform decisions about the effectiveness of an intervention in usual practice and thus, they should adopt designs that emphasize external validity and minimally deviate from routine care.^1^ Explanatory trials, on the other hand, are designed with the intent to generate understanding about the mechanism of action of the intervention and thus involve tightly controlled experimental conditions that facilitate the isolation of the aspect of interest. In recent years, interest in pragmatic trials has increased among funders, researchers, patients, and health system stakeholders.^2,3^ In part, this interest has been motivated by a recognition that results obtained in explanatory trials may not be replicated when the intervention is adopted into routine clinical practice.^4^ Moreover, the cost and logistical complexity of traditional explanatory trials have motivated funders and researchers to identify more cost-efficient approaches, for example, by leveraging existing infrastructure such as health administrative data.^5^

Despite their potential strengths, pragmatic trials pose challenges to existing ethical and regulatory frameworks, which were not developed with pragmatic trials in mind.^6^ In particular, traditional written informed consent has been identified as a key topic of discussion:^7,8^ some argue that standard written informed consent requirements may not be appropriate for low-risk pragmatic trials because the informed consent process may negatively impact trial recruitment, which in turn, may reduce the generalisability of the results and thereby undermine the pragmatic aim of the trial.^9,10,11,12^ Accordingly, some have argued for an expanded use of waivers of consent, while others have proposed alternative approaches to standard written consent.^13,14^ Regardless of the consent approaches used, the International Committee of Medical Journal Editors (ICMJE) requires explicit reporting of this protection in reports of randomised trials.^15^

While recent reviews have examined the use of waivers of consent in cluster randomised trials,^16^ noting that pragmatism was invoked as a rationale for the use of waivers in some studies, no reviews have examined whether arguments advanced in the literature have taken hold in the field by formally examining associations between pragmatism and use of waivers of consent in a broad sample of trials. A recent study suggested that pragmatic trials may be more likely to report a waiver of consent;^17^ however, this study focused only on highly cited trials and included a relatively small number of pragmatic trials. Moreover, this study did not explore whether specific trial characteristics were associated with the use of waivers of consent. Furthermore, to our knowledge, no previous studies have examined the prevalence of use of alternative approaches to standard written informed consent (such as integrated consent) that have been specifically proposed for pragmatic trials.

Our main objectives in this review are to describe the reporting of informed consent in a broad sample of pragmatic trials published between 2014 and 2019, and to identify characteristics associated with reporting and obtaining consent. Specific objectives are to determine:

1. What proportion of pragmatic trials include a statement about informed consent in the report? What proportion indicate that a waiver of consent had been obtained? If waivers of consent had been obtained, what justifications are provided?
2. What is the prevalence of reporting alternative consent approaches in pragmatic trials?
3. How do the reporting and obtaining of consent vary over time and by characteristics such as country, setting, trial design, type of study intervention, and self-identification as a pragmatic trial?

## METHODS

### Search strategy and identification of trials

The search strategy and identification of trials, as well as a descriptive account of the trials included in the review, have been previously published.^18^ In short, a published electronic search filter (Supplemental Table S1) was used to identify reports of trials more likely to be pragmatic.^19^ We implemented the search in Ovid MEDLINE to cover the period from 1 January 2014 to 3 April 2019. We included primary reports of randomised controlled trials in health research. Inclusion and exclusion criteria are summarised in Supplemental Table S2. Among 4337 eligible primary trial reports, 1988 reported trial registration in ClinicalTrials.gov (CT.gov) and are the focus of this review. We focused on these trials to facilitate the use of trial descriptors downloaded from CT.gov in our analysis.

### Data elements for extraction

Study start date, intervention type (classified in the registry as behavioral, drug, device, procedure, biological, dietary supplement, radiation, diagnostic, combination, genetic, other), and primary purpose (classified as treatment, prevention, health services research, supportive care, diagnostic, screening, other), were downloaded from CT.gov.^20^ Journal impact factors were obtained from Journal Citation Reports 2018 (JCR), and country of corresponding author from Web of Science. A standardized extraction form was developed to extract additional trial characteristics and consent items from the full text. We extracted the country/region of study recruitment and classified the setting as either public health or clinical. We classified each trial based on whether the word “pragmatic” was used to describe the trial, and the design as either individual or cluster randomisation. For cluster randomised trials, we classified interventions as cluster-cluster, professional-cluster, external-cluster, or individual-cluster, following Eldridge and colleagues.^21^ Cluster-cluster interventions were defined as any interventions delivered to the entire cluster and thus, not divisible at the individual level (e.g., posters placed in waiting rooms, mass media campaigns). Professional-cluster interventions were defined as any interventions delivered to health professionals (e.g., education or audit and feedback). External-cluster interventions were defined as interventions requiring individuals to be seen by staff external to the cluster, such as specialist nurses. Individual-cluster interventions were defined as any interventions delivered directly to individual participants (e.g., vaccines, patient educational leaflets). Because cluster randomised trials frequently evaluate complex interventions, each trial could be classified using multiple selections.

We extracted whether a statement about informed consent at the individual level was reported and if not, whether any justification for not obtaining consent or obtaining a waiver of consent was provided. We noted explicit reference to alternative consent approaches that have been proposed for pragmatic trials: electronic consent, simple opt-out, integrated consent, or shortened consent forms.^13,14^ Electronic consent was classified as any reference to electronic tablets and internet applications used in the consent process; integrated consent was classified as any reference to “clinical-style consent” or a brief verbal consent similar to what would occur normally during a clinical interaction between a physician and their patient;^14^ shortened consent form was classified as any statement about the consent form being simplified or shortened; simple opt-out was classified as any reference to potential participants being told they will be included in the research unless they decline. We also extracted what the consent or waiver of consent was for (e.g., trial participation, receiving an intervention, data collection); and whether the report referenced supplemental material wherein consent forms could be found.

The extraction form was first pilot tested and applied to a random sample of trials by five reviewers (JZZ, MT, SGN, KC, HPN) for training purposes. Once reviewers were considered adequately trained (this point was reached after 43 trials), the remaining trials were divided among the five reviewers who independently extracted data from each trial. The extractors met regularly to discuss any trials that raised difficulties for interpretation and consulted with CEG and CW whenever necessary.

### Missing data

When trial descriptors downloaded from CT.gov were missing, the trial report was reviewed to retrieve the missing information. Impact factors for a small number of journals were not available. To avoid excluding these studies from the multivariable analyses, we imputed values from the SCImago Journal & Country Rank (SJR).^22^

### Analysis

Data were summarized using frequencies and percentages for categorical variables, and range, median, and interquartile range (Q1–Q3) for continuous variables. Cross-tabulations with chi-squared tests of association and Cochran-Armitage trend tests were used to describe variation in reporting and obtaining informed consent across trial characteristics. To analyze factors associated with reporting on consent, we compared studies that included any statement about consent (whether obtained or not) with studies that did not include any statement about consent. To analyze factors associated with obtaining consent, we compared studies which indicated that consent had been obtained with studies that either indicated no consent or did not state anything about consent; thus, we combined trials that did not report consent with trials that explicitly stated they did not obtain consent or had obtained a waiver of consent. This was thought to be appropriate as it is likely that if consent had been obtained, authors would have stated so.^23^ A sensitivity analysis to explore the potential implications of this assumption was conducted by excluding trials from the analysis if they did not include any statement about consent. For a small number of trials that reported consent for one aspect of the trial (e.g., data collection) but a waiver of consent for another aspect (e.g., study intervention), we decided to classify the trial according to whether or not consent was obtained for the study intervention.

The trial characteristics of interest in these tests of association were pre-specified (rationales for each characteristic are summarised in Supplemental Table S3): publication year, clinical trial start year, journal impact factor (in tertiles), setting, trial design (cluster vs. individual randomisation), self-identification as pragmatic, country/region of study conduct, country/region of corresponding author, intervention type, and primary purpose. Some trial characteristics (e.g., type of intervention) had too many categories relative to the number of trials and categories were combined prior to analysis. For cluster randomised trials, many trials had multiple intervention components. We therefore classified each trial into one of three mutually exclusive categories following Eldridge and colleagues:^21^ trials with neither professional-level nor cluster-level interventions (i.e., trials in which individual consent was likely possible); trials with at least some professional-level interventions but no cluster-level interventions; and trials with at least some cluster-level interventions (i.e., trials in which individual consent may not have been possible because the intervention was not divisible at the individual level). We then conducted multivariable logistic regression analyses to examine the independent associations of characteristics of interest in the presence of the others, reporting odds ratios (ORs) and 95% confidence intervals (CIs). Variance inflation factors were used to rule out multi-collinearity. All analyses were performed using SAS, V.9.4 (SAS Institute, Cary, North Carolina, US) and the level of significance was set at α=0.05.

### Research ethics approval

This study did not involve human research participants and research ethics approval was not required.

## RESULTS

### Trial characteristics

Table 1 presents descriptive characteristics of the 1988 included trials. Trials were published across 524 different journals with impact factors ranging from 0.11 to 70.67 (median 4.95). Clinical trial start year ranged from 1998 to 2018. The most common countries/regions of study conduct were the United States (US) and the European Union (EU). Close to one-third (688/1988) were cluster randomised; the rest were individually randomised. Among the 688 cluster randomised trials, 278 (40.4%) had no professional-level or cluster-level interventions; 127 (18.5%) had at least some professional-level interventions but no cluster-level interventions; and 283 (41.1%) had at least some cluster-level interventions.

### Reporting of informed consent

Table 2 describes the reporting of informed consent and the justifications provided for no consent or waivers of consent. A total of 1856 (93.4%) reports included a statement about informed consent: of these, 1691 (91.1%) reported consent had been obtained, 139 (7.5%) reported consent had not been obtained or a waiver had been obtained, and 26 (1.4%) reported consent had been obtained for one aspect (e.g., data collection) but not for another (e.g., being exposed to an intervention). Of the 165 trials that reported not obtaining consent or obtaining a waiver of consent for at least one aspect of the trial, 76 (46.1%) provided a justification. Out of the 76 trials with justifications, 22 (28.9%) cited minimal risk, 10 (13.2%) cited infeasibility, and 5 (6.6%) cited that the waiver would not negatively impact the rights or welfare of participants. A variety of other justifications were provided that are not in line with international guidelines, including that the treatments were standard of care or followed current guidelines (22, 28.9%), the trial involved routine data collection (7, 9.2%), and pragmatism or the need to improve generalizability (6, 7.9%). Alternative consent approaches were reported in 53 (2.9%) with 37 (17.9%) referring to electronic consent, 14 (6.8%) to simple opt-out, 2 (1.0%) to integrated consent, and 1 (0.5%) to shortened consent forms. The details on the aspects (e.g., randomisation, study interventions, or data collection) that consent was obtained for are presented in Supplemental Table S4. Fourteen trials (0.7%) referenced a website or supplementary material where the consent documents can be found.

### Characteristics associated with not reporting on consent

Supplemental Table S5 describes variation in prevalence of not reporting consent across trial characteristics. Notably, cluster randomised trials (especially those with professional-level and at least some cluster-level interventions), and trials for which the primary purpose was health services research, had higher prevalence of not including a statement about consent. The results from the multivariable logistic regression analyses of factors associated with not reporting on consent are presented in Table 3. After controlling for all other factors, journal impact factor (p=0.001) and cluster randomisation (p<0.0001) were significantly associated with not reporting on consent.

### Characteristics associated with not obtaining consent

Supplemental Table S6 presents variation in prevalence of not obtaining consent across trial characteristics. More recent trials (starting between 2013 and 2018), cluster randomised trials (especially those with professional-level and at least some cluster-level interventions), trials self-identified as pragmatic, trials conducted in Canada only or US only, trials with non-clinical interventions, and trials for which the primary purpose was health services research, had higher prevalence of not obtaining consent. Results were similar when excluding trials that did not include any statement about consent (Supplemental Table S7).

The results from the multivariable logistic regression analyses to identify factors independently associated with not obtaining consent are presented in Table 4. Intervention type had to be excluded from consideration in these analyses as it was collinear with primary purpose. The results indicate that later trial start year, cluster randomisation, self-identification as pragmatic, higher income country settings, and trial purpose classified as health services research were significantly associated with not obtaining consent. Notably, the odds ratio of not obtaining consent among trials starting after 2013 versus before 2010 was 2.1 (95% CI 1.5 to 3.0), and among trials self-identified as pragmatic versus not was 2.1 (95% CI 1.6 to 2.9). Furthermore, the odds of not obtaining consent were higher among cluster randomised trials than individually randomised trials; even among cluster trials with no cluster-level or professional-level interventions, the odds of not obtaining consent were significantly higher than in individually randomised designs (OR=2.2, 95% CI 1.4 to 3.4).

## DISCUSSION

### Summary of principal findings

We found a high prevalence of *reporting* on informed consent but poor reporting of justifications for not *obtaining* consent. When justifications were provided, these were not in line with the minimum criteria stated in international ethics guidance.^24^ Not surprisingly, higher journal impact factor was associated with more complete reporting; cluster randomisation on the other hand, was significantly associated with not reporting on consent, which may reflect confusion among investigators about the need to report consent in more complex trial designs.

We found a relatively high prevalence of obtaining consent (85.0% compared to 7.0% reporting a waiver or no consent). Relative to the use of waivers, we were surprised to find few trials reporting alternative consent approaches, although we recognize that most trials were initiated before publication of proposals for alternative approaches to standard written consent. We were not surprised to find a lower prevalence of obtaining consent in cluster randomised trials as consent may be infeasible in the case of cluster-level or professional-level interventions; however, even among cluster trials with no cluster-level or professional-level interventions, the odds of not obtaining consent were still significantly higher than among individually randomised trials. Ethical guidance for the design and analysis of cluster randomised trials has been provided by the 2012 *Ottawa Statement on the Ethical Design and Conduct of Cluster Randomized Trials*.^25^ The *Ottawa Statement* recommends that consent be obtained from individual research participants unless a waiver of consent is granted by the research ethics committee. The *Ottawa Statement* endorses waivers of consent if the research is not feasible without the waiver (e.g., in the case of cluster-level interventions) and if the study interventions and data collection procedures pose no more than minimal risk. Individual-cluster trials with waivers of consent are attractive designs for advancing pragmatic research,^16^ but further work is required to determine whether the use of cluster randomisation warrants a different standard of consent than individual randomisation.^26^ Further work is also required to explore the use of a waiver for one aspect (e.g., intervention) but securing consent for another (e.g., data collection) as this raises the question of why consent was deemed impossible or impracticable. Independent of the design, trials that self-identified as pragmatic had higher prevalence of not obtaining consent than those that did not use this label. The issues posed by the standard written informed consent process for pragmatic trials have been discussed extensively,^10,11,13,27^ although pragmatism is not currently an accepted justification for not obtaining consent. The findings that trial recency and conduct in higher income country settings are associated with not obtaining consent may be related to the recent push for more pragmatic trials by funders in these countries. Another possible explanation is that higher income countries (e.g., US, UK, EU, Canada, Australia) have research regulations that allow for waivers or alterations, whereas some LMICs (e.g., South Africa) only permit waivers in very limited circumstances such as human tissue and genetic research, and others (e.g., Sierra Leone) do not permit waivers — perhaps justly so given that research participants in low-resource settings may be vulnerable to exploitation by sponsors and investigators from wealthier countries.^28^

### Comparison with other studies

Dhamanaskar and Merz^17^ reviewed a sample of 500 highly cited trials published between 2014 and 2018 and found that 8.8% did not secure informed consent from at least some participants with no evidence that the prevalence changed over time. Our review found a comparable percentage of no consent (8.8% or 14.8% — depending on whether the trials that did not include a statement about consent are included or excluded) but in contrast, our review found that there was an increase between 1998 to 2018 when using the date of trial initiation. Lin et al^29^ reported the prevalence of waivers of consent in 103 trials published in either 2014 or 2017. Trials were included if they were described as pragmatic or using pragmatic methods by the trial authors or described as comparative effectiveness trials, and involved at least one site in the US. They found that 22% did not obtain consent. When considering the comparable subset of self-labelled pragmatic trials conducted in the US in our sample, the percentage was 33.1% or 26.9% — depending on whether trials that did not include a statement about consent are included or excluded). Unlike our review, these authors found no significant change in the use of waivers of trials published in 2014 and 2017.

### Strengths and limitations

To our knowledge, this is the first review that aimed to describe the reporting of consent in a broad sample of pragmatic trials. The review examined not just the reporting of this ethical protection but further details such as justifications for waivers of consent, and the use of alternative consent approaches that have been proposed for pragmatic trials. In contrast to other studies that examined consent reporting in top journals or highly-cited publications,^17,30,31^ we examined the reporting of consent in trial reports published in 524 journals that span the range of journal impact factors.

Limitations of this study include the following: First, identification of pragmatic trials in the literature is challenging. Other reviews of pragmatic trials have used limited or arbitrary search terms to identify pragmatic trials. We used a published search filter to identify trials more likely to be pragmatic based on terms and phrases used in the title or abstract, including (but not limited to) the term “pragmatic”. An advantage of our approach is that, since no reporting guidelines require authors to label their trials as pragmatic in the title or abstract, our search likely captured a broader range of trials with pragmatic intention, as opposed to only those that explicitly use the term. Furthermore, our search retrieved trials that used the term in the full text but not in the title or abstract. Because of the noted challenges in retrospectively evaluating trial manuscripts with tools such as PRECIS-2,^32^ we did not score each trial individually to confirm that its design would be classified as pragmatic by this instrument. Even if retrospective scoring of a large database of trials were feasible, there is no objective threshold for determining when a trial can be considered sufficiently “pragmatic”. Second, the trials included in this review are the subset with a corresponding registration in CT.gov and therefore may not be representative of all pragmatic trials published in the literature. Registration in CT.gov is a US federally mandated requirement for clinical investigations with sites in the US and therefore trials included in our review may be more representative of US practices.^33^ Third, some of our variables were downloaded directly from CT.gov, thus any inaccurate classification of data in CT.gov may have influenced our findings. The classification of intervention type was heterogeneous with a relatively large proportion classified as “other”. Despite intervention type being an important factor associated with obtaining consent, it could not be considered in the multivariable model due to collinearity with primary trial purpose. Given potential ambiguity in how intervention type was classified in CT.gov, we chose to retain primary trial purpose in our model instead. Fourth, we relied only on information reported in the trial publication; however, reporting practices may not accurately reflect trial conduct and thus, our study may have been subject to misclassification. Finally, the brief descriptions of consent procedures in trial manuscripts do not provide the level of detail that is required to fully describe alternative consent approaches and to fully understand the reasons and context behind waivers and alterations of consent. Integrated consent, for example, is difficult to differentiate from other forms of verbal consent. As such, we may have under-estimated the use of alternative consent approaches.

## CONCLUSION

Pragmatic trials are diverse and raise ethical challenges that have not yet been adequately addressed. We found increasing prevalence of not obtaining consent which may be linked to explicit goals of pragmatism. Whilst our review is unable to conclude that waivers of consent were inappropriate, the low frequency of trials reporting on alternative approaches to consent specifically proposed for pragmatic trials is a notable finding. A choice between either a waiver of consent or standard written informed consent may reflect a false dichotomy on the part of research ethics committees and investigators: greater attention to alternative consent approaches, when justified and approved by a research ethics committee, may facilitate the conduct of more pragmatic trials while preserving the autonomy of prospective research participants and the trust of patients and the public in research. We recommend that journal editors and peer reviewers demand that details about consent are made explicit in published trial reports and justifications are provided when consent was not sought.

## Supplementary Material

Supp1

## Figures and Tables

**Table 1:** Descriptive characteristics of trials included in the review (N = 1988) (Created by the authors)

Descriptive item	Frequency (%) unless otherwise indicated
**Publication year**	
2014–2015	639 (32.1)
2016–2017	760 (38.2)
2018–2019	589 (29.6)
**Journal Impact Factor**^a^ **(N = 1984)**	
Median (Q1–Q3)	4.95 (2.98 – 14.15)
Min, Max	0.11, 70.67
**Clinical trial start year** ^b^	
1998–2010	722 (36.3)
2011–2012	583 (29.3)
2013–2018	683 (34.4)
**Years from clinical trial start date to publication**	
Median (Q1–Q3)	5 (4 – 6)
Min, Max	0, 18
**Country of corresponding author** *	
United States (US)	993 (49.9)
European Union (EU)	436 (21.9)
Canada	119 (6.0)
United Kingdom (UK)	106 (5.3)
Australia or New Zealand	18 (0.9)
Other developed country	132 (6.6)
Low- and Middle-Income Country (LMIC)	221 (11.1)
**Country of study conduct** *	
United States (US)	866 (43.6)
European Union (EU)	458 (23.0)
Canada	140 (7.0)
United Kingdom (UK)	93 (4.7)
Australia or New Zealand	31 (1.6)
Other developed country	268 (13.5)
Low- and Middle-Income Country (LMIC)	353 (17.8)
Not reported or Unclear	20 (1.0)
**Setting**	
Public health	417 (21.0)
Clinical	1571 (79.0)
**Intervention type** ^c^	
Behavioral	864 (43.5)
Drug	296 (14.9)
Device	130 (6.5)
Procedure	106 (5.3)
Biological	38 (1.9)
Dietary Supplement	36 (1.8)
Radiation	3 (0.2)
Diagnostic	3 (0.2)
Combination Product	3 (0.2)
Genetic	1 (0.1)
Other	508 (25.6)
**Primary purpose** ^d^	
Treatment	766 (38.5)
Prevention	515 (25.9)
Health Services Research	357 (18.0)
Supportive Care	175 (8.8)
Diagnostic	67 (3.4)
Screening	58 (2.9)
Other	50 (2.5)
**Self-identified as pragmatic in full text?**	
Yes	419 (21.1)
No	1569 (78.9)
**Trial design**	
Individually randomised	1300 (65.4)
Cluster randomised	688 (34.6)
Types of interventions in cluster randomised trials* **(N = 688)**	
Individual-cluster	388 (56.4)
Professional-cluster	250 (36.3)
Cluster-cluster	283 (41.1)
External-cluster	53 (7.7)
**Mutually exclusive groupings of cluster randomised trial, based on types of interventions (N = 688)**	
No professional-level or cluster-level interventions	278 (40.4)
Professional-level intervention, possibly other interventions, no cluster-level interventions	127 (18.5)
At least some cluster-level interventions	283 (41.1)

* Does not sum to 100% as a single study can belong to multiple categories.

a Impact factors for 65 trials were imputed using the SCImago Journal & Country Rank or Google search as of January 2, 2021; impact factors could not be found for 4 trials.

b CT.gov data missing for 7 trials and were obtained from the manuscript.

c CT.gov data missing for 14 trials and classified using information from the manuscript.

d CT.gov data missing for 77 trials and classified using information from the manuscript.

**Table 2:** Reporting of informed consent and waivers of informed consent (N = 1988) (Created by the authors)

Item	Frequency (%)
**Statement about individual informed consent included in the report?**	
Yes	1856 (93.4)
No	132 (6.6)
**Type of statement included (N = 1856)**	
Consent reported as obtained	1691 (91.1)
Consent reported as not obtained or waiver obtained	139 (7.5)
Mixture: Consent obtained for one aspect but not obtained for another	26 (1.4)
**Justification provided for no consent or waiver of consent? (N = 165)**	
Yes	76 (46.1)
No	89 (53.9)
**Type of justification provided**^a^ **(N = 76)**	
Study involved minimal risk	22 (28.9)
Obtaining consent would have made the study impossible or infeasible	10 (13.2)
Waiver would not negatively affect rights or welfare of participants	5 (6.6)
Treatment or study procedure was standard of care or followed current guidelines	22 (28.9)
Trial involved routine data collection	7 (9.2)
To conduct trial in a pragmatic manner and/or to improve generalizability	6 (7.9)
Intervention was at the cluster-level	6 (7.9)
Trial was classified as quality improvement	6 (7.9)
Consent would bias the response or the results	5 (6.6)
Emergency research	4 (5.3)
Intervention only targeted the health system and health-care workers or no direct patient contact	3 (3.9)
Intervention not clinical	3 (3.9)
De-identified data or data could not be traced back to individuals	3 (3.9)
Other non-specific justification	11 (14.5)
No explicit justification provided other than citing legislation	5 (6.6)
**Explicit reporting of any alternative consent approaches? (N = 1856)**	
Yes^a^	53 (2.9)
Electronic consent	37
Simple opt-out^b^	14
Integrated consent	2
Shortened consent form	1
No	1803 (97.1)

a Does not sum to 100% as trial can have more than one type of approach.

b Of these trials, 5 were characterized by trial authors as having obtained consent, whereas 9 were characterized by trial authors as not having obtained consent.

**Table 3:** Results from the multivariable logistic regression analysis to identify independent characteristics associated with not reporting on consent (Created by the authors)

Trial characteristics*	Adjusted OR (95% Confidence Interval)	p-value
**Publication year**		0.289
2014 – 2015	Ref	
2016 – 2017	1.05 (0.68 to 1.61)	0.835
2018 – 2019	0.73 (0.45 to 1.19)	0.209
**Journal impact factor**		0.001
Tertile 1: [0.11 – 3.575)	Ref	
Tertile 2: [3.575 – 7.958)	1.34 (0.88 – 2.05)	0.168
Tertile 3: [7.958 – 70.67]	0.53 (0.32 – 0.88)	0.015
**Design**		<0.0001
Individually randomised trial	Ref	
CRT with no professional-level or cluster-level interventions	1.58 (0.84 – 2.97)	0.156
CRT with professional-level intervention, possibly other interventions, no cluster-level interventions	7.84 (4.68 – 13.12)	<0.0001
CRT with at least some cluster-level interventions	5.25 (3.30 – 8.34)	<0.0001
**Country of corresponding author**		0.213
LMIC only	Ref	
Other	0.47 (0.16 to 1.42)	0.183
EU only or UK only	1.24 (0.60 to 2.56)	0.561
Canada only or US only	1.26 (0.64 to 2.47)	0.508
**Setting**		0.152
Clinical	Ref	
Public health	0.70 (0.43 – 1.14)	0.152
**Self-identified as pragmatic**		0.249
No	Ref	
Yes	1.29 (0.84 – 1.99)	0.249

* Only pre-specified characteristics were entered into the model.

CRT = Cluster randomised trial

**Table 4:** Results from the multivariable logistic regression analysis to identify independent characteristics associated with not obtaining consent (Created by the authors)

Trial characteristics*	Adjusted OR (95% Confidence Interval)	p-value
**Start year**		<0.0001
1998 – 2010	Ref	
2011 – 2012	1.24 (0.85 – 1.80)	0.260
2013 – 2018	2.13 (1.52 – 3.00)	<0.0001
**Journal impact factor**		0.569
Tertile 1: [0.11 – 3.575)	Ref	
Tertile 2: [3.575 – 7.958)	1.09 (0.78 – 1.53)	0.608
Tertile 3: [7.958 – 70.67]	0.91 (0.64 – 1.30)	0.587
**Design**		<0.0001
Individually randomised trial	Ref	
CRT with no professional-level or cluster-level interventions	2.17 (1.40 – 3.37)	0.0005
CRT with professional-level intervention, possibly other interventions, no cluster-level interventions	5.09 (3.22 – 8.04)	<0.0001
CRT with at least some cluster-level interventions	8.09 (5.63 – 11.64)	<0.0001
**Country of study conduct**		<0.0001
LMIC only	Ref	
Other	2.31 (1.26 – 4.20)	0.006
EU only or UK only	2.54 (1.47 – 4.37)	0.0008
Canada only or US only	4.00 (2.46 – 6.51)	<0.0001
**Setting**		0.073
Clinical	Ref	
Public health	0.70 (0.48 – 1.03)	0.073
**Self-identified as pragmatic**		<0.0001
No	Ref	
Yes	2.14 (1.57 – 2.91)	<0.0001
**Primary purpose**		<0.0001
Treatment	Ref	
Other	1.35 (0.93 – 1.96)	0.118
Health services research	3.15 (2.13 – 4.67)	<0.0001

* Only pre-specified characteristics were entered into the model. Intervention type (Clinical, Dietary and behavioral, Other) was excluded as it was collinear with other variables.

CRT = Cluster randomised trial
