# Supplementary material for: Informed Consent in Pragmatic Trials: Results from a Survey of Trials published 2014–2019
Source: J Med Ethics. Author manuscript; Available in PMC 2023 May 15. (PMC9107524; doi:10.1136/medethics-2021-107765)
Supplement: Supp1 [file NIHMS1757155-supplement-Supp1.docx]

**SUPPLEMENTAL MATERIAL**

**Table S1: Electronic search filter to identify pragmatic trials in MEDLINE (created by the authors)**

| **#** | **Search Statement** |
| --- | --- |
|  | **Trial design terms** |
| 1 | (((pragmatic$ OR naturalistic OR real world OR real life OR unblinded OR unmasked OR cluster OR step$ wedge$ OR point of care OR factorial OR switchback OR switch back OR phase 4 OR phase IV) adj10 (study OR trial)) OR (practical trial OR effectiveness trial OR ((cluster$ or communit$) adj2 randomi$))).tw. |
|  | **Trial attribute terms** |
| 2 | (general practice$ OR primary care OR registry based OR health record$ OR medical record$ OR EHR OR EMR OR administrative data OR routinely collected data OR (communit$ adj2 intervention$) OR quality improvement OR implementation OR decision support OR health service$ OR health system$ OR comparative effectiveness OR CER OR usual care OR evidence based OR practice guideline$ OR (guideline$ adj1 recommend$) OR knowledge translation OR health technology assessment OR HTA OR cost effectiveness OR process evaluation OR economic evaluation OR patient oriented).tw. |
|  | **Limit to records likely to be RCTs** |
| 3 | randomized controlled trial.pt. OR ((comparative effectiveness OR randomi?ed) adj10 (trial OR study)).ti. |
| 4 | (comment on OR phase 1 OR phase I OR phase 2 OR phase II OR non-randomi?ed OR quasi-randomi?ed OR pseudo-randomi?ed).ti. OR (clinical trial, phase I OR clinical trial, phase II OR systematic review OR meta-analysis OR review OR editorial).pt. |
|  | **Include records tagged as pragmatic trials** |
| 5 | pragmatic clinical trial.pt. |
|  | **Sensitivity-maximizing search (combines trial design terms or attribute terms with RCT terms)** |
| 6 | ((1 OR 2) AND (3 NOT 4)) OR 5 |
| 7 | exp Animals/ NOT Humans/ |
| 8 | 6 NOT 7 |

**RCT=randomized controlled trial**

**Table S2 Screening: Inclusion and exclusion criteria for creation of the large database of pragmatic trials (created by the authors)**

| **INCLUSION CRITERIA** | | **SPECIFICATION** |
| --- | --- | --- |
| **Health RCT** | A health RCT evaluates interventions aimed at changing subjective or objective measures of individual or group health status, or of processes which lead to changes in health status. Health status is defined as a state of human wellbeing, in individuals or groups, with physical or mental health correlates. | Trial must use randomization (as opposed to quasi-randomization) and must be comparative (i.e., at least one intervention and one control arm, or at least two intervention arms for “comparative effectiveness research”). |
| **Health care RCT** | A health care RCT is one aimed at evaluating changes in the delivery of services to changing health status, or to change processes in the delivery of care that are known to lead to changes in health status. This includes RCTs of treatment, prevention, health promotion, health knowledge or behaviour as well as studies of the implementation, acceptability, efficiency, equity or cost of interventions for treatment, prevention, promotion, health knowledge or behaviour change.  These studies may target individual patients, groups of patients, communities or populations, and/or carers from family, community and health care systems. | Trial must have a target enrolment of at least 100 individuals |
| **EXCUSION CRITERIA** | | **SPECIFICATION** |
| 1 | Not a randomized controlled trial | Trial must use randomization (as opposed to quasi-randomization) and must be comparative (i.e., at least one intervention and one control arm, or at least two intervention arms for “comparative effectiveness research”). |
| 2 | RCT but with <100 target enrolment | Trial must have a target enrolment of at least 100 individuals |
| 3 | Study protocol or design paper | Study does not report trial outcomes or reports only baseline data. Includes study protocols and published statistical analysis plans. |
| 4 | Methods paper – not a trial report | Exclude if the article doesn’t present any results from a trial, e.g., it may only describe the design of a trial without presenting results or it may talk about recruitment difficulties or intervention development only or only a statistical analysis plan. Some studies may be a trial themselves e.g. the trial is about the best consent approach within a trial and they test two consent methods. |
| 5 | Not health research | Excluded trials include those of purely educational intervention in school-based settings that don’t have a distinct health link. For example, a trial testing two different Mathematics curricula or trials assessing interventions to reduce bullying, but there is no direct health link (e.g., does not involve school nurses and does not assess health outcomes, e.g., anxiety.) There must be a clear link to health research. Other exclusion examples include trials testing new ways of sweeping the floor, or new administrative approaches of contacting patients, or recruiting patients into trials, or improving data quality, or new hiring practices. Unless there was a clear and direct route by which this would impact on patient health outcomes, the trial is not eligible (lack of direct relevance to patient health). |
| 6 | Pilot or feasibility study | If the trial is obviously labelled as a pilot or feasibility study, it is ineligible. If the conclusions simply refer to “feasibility”, but also gives results for a primary health or health care outcome and it meets all the other criteria for inclusion the trial would be included. |
| 7 | Not a pragmatic intent | Trial that is obviously not of pragmatic orientation. For example, trials that focused on isolating a biological impact of an intervention without a clear clinical implication, or that did not assess clinical outcomes, were deemed more likely to not to have a pragmatic orientation. |
| 8 | Non-primary trial report | Any indication in the manuscript that it is not the primary trial report (meaning, the analysis of the primary trial outcome), indicates exclusion. Examples of non-primary reports include the analysis of an outcome clearly identified as a secondary trial outcome; subgroup analysis with primary trial results reported elsewhere; long-term follow-up (with primary endpoint reported previously), process analysis, mediation analysis, sensitivity analysis (if reported separately to primary outcome).** |
| 9 | Educational intervention of health professionals with no real patients or patient data | Studies randomizing clinicians to different vignettes and then surveying them to assess their response are excluded as they do not involve real patients. Other trials excluded here are: trials involving only manikins or simulations (e.g., for training providers to do CPR) or trials of purely educational (provider) interventions which do not measure patient data. For example, if a trial tests two different Medical curricula or outcomes are exclusively measures of clinical knowledge measured in clinicians or medical residents, the trial is not eligible. |
| 10 | Other (specify) |  |

** Trial registration information was used to facilitate identification of the primary trial report from among multiple publications from the same trial. Studies were also compared by first and senior authors and studies that reported the same trial were flagged. Articles flagged as potential non-primary reports were also scrutinized for any explicit statement referring to “primary results being published previously” or that the present article was presenting a secondary analysis. When multiple publications were associated with the same clinical trial registration, a decision had to be made about whether any could be considered the primary trial publication. In cases of uncertainty, the primary outcome in the registry was used to guide the decision. In the case of complex study designs, such as factorial designs with interventions reported separately, the article reporting on the first listed intervention or outcome reported in the registry was selected as the primary report. Finally, our search identified several Health Technology Assessment (HTA) reports, unique to studies funded by the UK National Institutes of Health Research. When the HTA report was the only report of the trial retrieved it was retained as the primary publication; otherwise, the associated journal publication of the trial was retained as the primary publication.

**Table S3: Rationale and grouping of trial characteristics for analyses of factors associated with reporting and obtaining consent (created by the authors)**

| **Trial Characteristics^a,b^** | **Categories** | **Grouping Rationale** | **Rationale for inclusion in analysis** |
| --- | --- | --- | --- |
| Publication year | 3 categories   - 2014-2015 - 2016-2017 - 2018-2019 | Grouped to ensure approximately even numbers of trials in each category; we attempted to create more meaningful groupings, for example, by considering the timing of the trial start year relative to a major change in research ethics guidelines, however, this did not lead to feasible groupings (e.g., the Council for International Organizations of Medical Sciences (CIOMS) made changes to the International ethical guidelines for health-related research involving humans in 2016^1^ and too few trials started on or after this date). | Reporting of consent may have improved over time. |
| Clinical trial start year | 3 categories   - 1998-2010 - 2011-2012 - 2013-2018 |  | The conduct of consent may have changed over time, for example, due to the introduction of new regulations or changes in existing regulations, or evolving beliefs and accepted scientific practices about the need for consent. |
| Journal impact factor | 3 categories   - Lowest tertile - Middle tertile - Highest tertile | Grouped into tertiles to simplify interpretation. | Quality of reporting may vary by journal impact factor and higher and lower impact factor journals may have different review standards. |
| Setting | 2 categories   - Public health - Clinical | No further grouping done. | Authors doing public health research and those doing clinical research may adhere to different principles when they report. |
| Trial design | 4 categories   - Individually randomised trial - No professional-level or cluster-level interventions (i.e., only individual-cluster or external-cluster interventions) - Professional-level intervention, possibly other interventions, no cluster-level interventions - At least some cluster-level interventions | Used mutually exclusive grouping of typology types as per Eldridge and colleagues.^2^ This typology categorizes trials based on the feasibility of obtaining consent. For example, cluster randomised trials that involve no professional-level or cluster-level interventions may still be able to obtain consent because the target of the intervention is at the individual level. However, trials involving at least some cluster-level interventions are less likely to obtain consent for interventions because the intervention is not divisible at the individual level. | Reporting of consent in cluster randomised trials is more complicated for several reasons, including possible confusion about who should be considered the research participants and whose consent is required.  Consent practices are likely to vary according to the level of the intervention: individually randomised trials or cluster randomised trials with individual-level interventions may be more likely to obtain consent, compared to trials with cluster-level interventions where consent is more difficult. There is confusion surrounding who are considered the research participants in cluster randomised trials and individual consent may be less likely when using cluster randomisation, even when the interventions are administered at the individual level. |
| Self-identified as pragmatic | 2 categories   - Yes - No | No further grouping done. | Trials that self-identify as pragmatic may be less likely to obtain consent than trials that do not self-identify as pragmatic. |
| Country of study conduct | 4 categories   - Canada only or US only - EU only or UK only - LMIC only - Other | Grouped to compare consent between trials conducted in Canada or US only versus trials conducted in the EU or UK only versus trials conducted in LMICs only. We classified trials that were conducted in countries other than North America, EU, and LMICs and in multiple countries as other. | Consent practices and reporting of consent may vary by country because different countries have different regulations pertaining to requirements for consent. |
| Country of corresponding author | 4 categories   - Canada only or US only - EU only or UK only - LMIC only - Other |  | Consent practices and reporting of consent may vary by country of corresponding author as authors could be influenced by the accepted scientific practices and regulations of their home country. |
| Intervention type | 3 categories   - Clinical (Drug, Device, Biological, Procedure, Radiation, Genetic, Combination Product, and Diagnostic Test) - Dietary and behavioral (Dietary Supplement, Behavioral) - Other | Grouped into clinical, dietary and behavioral, and other. We left the intervention type other as a category on its own as the interventions of trials classified under this category do not fit the previous two categories based on a high-level review of the intervention descriptions for trials in this category. | Trials evaluating a clinical intervention may be more likely to be regulated by a regulatory agency.^3^ The consent requirements are also laid out in international guidelines that govern human research such as the International Council for Harmonisation of Technical Requirements of Pharmaceuticals for Human Use (ICH) Guidance E6(R2): Good Clinical Practice (ICH GCP).^4^ Consent is more likely to be obtained for these trials as the risks to the research participants may be higher compared to the other categories. |
| Primary purpose | 3 categories   - Treatment - Health Services Research - Other (Prevention, Supportive Care, Diagnostic, Screening, Basic Science, Educational/ Counseling/Training, Other) | Grouped into treatment, health services research, and other. We left the original categories of treatment and health services research as distinct categories as the former was the most prevalent purpose while the latter is a common type of research for pragmatic trials. | Trials with a treatment purpose are more likely to be scrutinized than trials with a health services research or other purpose. Consent is more likely to be obtained for treatment trials as the risks may be higher compared to health services research. |

^a^ Trial characteristics pre-specified in the multivariable logistic regression model for not reporting on consent: Publication year, Journal impact factor, Setting, Trial design, Self-identified as pragmatic, Country of corresponding author

^b^ Trial characteristics pre-specified in the multivariable logistic regression model for not obtaining consent: Clinical trial start year, Journal impact factor, Setting, Trial design, Self-identified as pragmatic, Country of study conduct, Intervention type, Primary purpose

**Table S4: Reporting of explicit details pertaining to type and purpose of consent for individually randomised trials and cluster randomised trials (created by the authors)**

|  | **Individually randomised trials** | **Cluster randomised trials** | **All trials** |
| --- | --- | --- | --- |
| **Item** | **N (%)** | **N (%)** | **N (%)** |
| **Explicitly reported that consent obtained was *written* consent?** | **N = 1205** | **N = 512** | **N = 1717** |
| Yes | 940 (78.0) | 353 (68.9) | 1293 (75.3) |
| No | 265 (22.0) | 159 (31.1) | 424 (24.7) |
| **Provided explicit details about what consent was for?^a^** | **N = 1205** | **N = 512** | **N = 1717** |
| Yes | 267 (22.2) | 198 (38.7) | 465 (27.1) |
| No | 938 (77.8) | 314 (61.3) | 1252 (72.9) |
| **Type of details provided*** | **N = 267** | **N = 198** | **N = 465** |
| Consent was for trial participation | 217 (81.3) | 123 (62.1) | 340 (73.1) |
| Consent was for data collection | 46 (17.2) | 77 (38.9) | 123 (26.5) |
| Consent was for study intervention | 15 (5.6) | 16 (8.1) | 31 (6.7) |
| Consent was for randomisation | 12 (4.5) | 0 (0.0) | 12 (2.6) |
| Consent was for another aspect of the trial (e.g., *continued* participation) | 10 (3.7) | 2 (1.0) | 12 (2.6) |
| **Provided explicit details about what waiver was for?^a^** | **N = 60** | **N = 105** | **N = 165** |
| Yes | 11 (18.3) | 15 (14.3) | 26 (15.8) |
| No | 49 (81.7) | 90 (85.7) | 139 (84.2) |
| **Type of details provided*** | **N = 11** | **N = 15** | **N = 26** |
| Waiver was for trial participation | 2 (18.2) | 3 (20.0) | 5 (19.2) |
| Waiver was for data collection | 4 (36.4) | 6 (40.0) | 10 (38.5) |
| Waiver was for study intervention | 3 (27.3) | 6 (40.0) | 9 (34.6) |
| Waiver was for randomisation | 3 (27.3) | 0 (0.0) | 3 (11.5) |
| Waiver was for another aspect of the trial | 0 (0.0) | 0 (0.0) | 0 (0.0) |
| **Reference informed consent documents in the report?** | **N = 1300** | **N = 688** | **N = 1988** |
| Yes | 10 (0.8) | 4 (0.6) | 14 (0.7) |
| No | 1290 (99.2) | 684 (99.4) | 1974 (99.3) |

* Does not sum to 100% as trial can have more than one type of detail.

^a^ We extracted details of consent or waiver reporting specific to participation, data collection, intervention, or randomisation or a related aspect (e.g., *continued* participation).

**Table S5: Variation in prevalence of not reporting on consent across trial characteristics (N = 1988) (created by the authors)**

|  | **Reported on consent?** | |  |
| --- | --- | --- | --- |
|  | **No**  **(N = 132)** | **Yes**  **(N = 1856)** | **p-value** |
| **Publication year** |  |  | 0.320 |
| 2014-2015 | 44 (6.9) | 595 (93.1) |  |
| 2016-2017 | 56 (7.4) | 704 (92.6) |  |
| 2018-2019 | 32 (5.4) | 557 (94.6) |  |
| **Clinical trial start year** |  |  | 0.304 |
| 1998-2010 | 48 (6.6) | 674 (93.4) |  |
| 2011-2012 | 29 (5.0) | 554 (95.0) |  |
| 2013-2018 | 55 (8.0) | 628 (92.0) |  |
| **Journal impact factor^a^** |  |  | 0.027 |
| Tertile 1: [0.11 – 3.575) | 46 (7.0) | 616 (93.0) |  |
| Tertile 2: [3.575 – 7.958) | 60 (9.1) | 603 (90.9) |  |
| Tertile 3: [7.958 – 70.67] | 26 (3.9) | 637 (96.1) |  |
| **Setting** |  |  | 0.879 |
| Public health | 27 (6.5) | 390 (93.5) |  |
| Clinical | 105 (6.7) | 1466 (93.3) |  |
| **Trial design** |  |  | <0.0001 |
| Individually randomised | 47 (3.6) | 1253 (96.4) |  |
| Cluster randomised | 85 (12.3) | 603 (87.7) |  |
| **Type of interventions in cluster trials (N = 688)** |  |  | <0.0001 |
| No professional-level or cluster-level interventions | 14 (5.0) | 264 (95.0) |  |
| Professional-level intervention, possibly other interventions, no cluster-level interventions | 29 (22.8) | 98 (77.2) |  |
| At least some cluster-level interventions | 42 (14.8) | 241 (85.2) |  |
| **Self-identified as pragmatic?** |  |  | 0.172 |
| Yes | 34 (8.1) | 385 (91.9) |  |
| No | 98 (6.3) | 1471 (93.7) |  |
| **Country of study conduct** |  |  | 0.018 |
| Canada only or US only | 73 (8.1) | 828 (91.9) |  |
| EU only or UK only | 31 (7.1) | 406 (92.9) |  |
| LMIC only | 17 (5.4) | 298 (94.6) |  |
| Other^b^ | 11 (3.3) | 324 (96.7) |  |
| **Country of corresponding author** |  |  | 0.113 |
| Canada only or US only | 80 (7.3) | 1013 (92.7) |  |
| EU only or UK only | 36 (7.0) | 479 (93.0) |  |
| LMIC only | 11 (5.7) | 181 (94.3) |  |
| Other^b^ | 5 (2.7) | 183 (97.3) |  |
| **Intervention type^c^** |  |  | 0.0008 |
| Clinical | 24 (4.1) | 556 (95.9) |  |
| Dietary and behavioral | 58 (6.4) | 842 (93.6) |  |
| Other | 50 (9.8) | 458 (90.2) |  |
| **Primary purpose^d^** |  |  | <0.0001 |
| Treatment | 25 (3.3) | 741 (96.7) |  |
| Health Services Research | 54 (15.1) | 303 (84.9) |  |
| Other | 53 (6.1) | 812 (93.9) |  |

^a^ Data for four trials were terminally missing and imputed the lowest journal impact factor of 0.11.

^b^ Other includes multinational trials.

^c^ Obtained from CT.gov and grouped as follows: Clinical (Drug, Device, Biological, Procedure, Radiation, Genetic, Combination Product, Diagnostic Test); Dietary Supplement and Behavioral; Other.

^d^ Obtained from CT.gov; Other includes Prevention, Diagnostic, Supportive Care, Screening, Basic Science, Educational/Counseling/Training, Other.

**Table S6: Variation in prevalence of not obtaining consent across trial characteristics (N = 1988) (created by the authors)**

|  | **Obtained consent?** | |  |
| --- | --- | --- | --- |
|  | **No^a^**  **(N = 295)** | **Yes**  **(N = 1693)** | **p-value** |
| **Publication year** |  |  | 0.359 |
| 2014-2015 | 92 (14.4) | 547 (85.6) |  |
| 2016-2017 | 107 (14.1) | 653 (85.9) |  |
| 2018-2019 | 96 (16.3) | 493 (83.7) |  |
| **Clinical trial start year** |  |  | <0.0001 |
| 1998-2010 | 79 (10.9) | 643 (89.1) |  |
| 2011-2012 | 78 (13.4) | 505 (86.6) |  |
| 2013-2018 | 138 (20.2) | 545 (79.8) |  |
| **Journal impact factor by tertile^b^** |  |  | 0.749 |
| Tertile 1: [0.11 – 3.575) | 95 (14.4) | 567 (85.6) |  |
| Tertile 2: [3.575 – 7.958) | 109 (16.4) | 554 (83.6) |  |
| Tertile 3: [7.958 – 70.67] | 91 (13.7) | 572 (86.3) |  |
| **Setting** |  |  | 0.169 |
| Public health | 53 (12.7) | 364 (87.3) |  |
| Clinical | 242 (15.4) | 1329 (84.6) |  |
| **Trial design** |  |  | <0.0001 |
| Individually randomised trial | 105 (8.1) | 1195 (91.9) |  |
| Cluster randomised trial | 190 (27.6) | 498 (72.4) |  |
| **Type of interventions in cluster trials (N = 688)** |  |  | <0.0001 |
| No professional-level or cluster-level interventions | 37 (13.3) | 241 (86.7) |  |
| Professional-level intervention, possibly other interventions, no cluster-level interventions | 44 (34.6) | 83 (65.4) |  |
| At least some cluster-level interventions | 109 (38.5) | 174 (61.5) |  |
| **Self-identified as pragmatic?** |  |  | <0.0001 |
| Yes | 103 (24.6) | 316 (75.4) |  |
| No | 192 (12.2) | 1377 (87.8) |  |
| **Country of study conduct** |  |  | <0.0001 |
| Canada only or US only | 175 (19.4) | 726 (80.6) |  |
| EU only or UK only | 60 (13.7) | 377 (86.3) |  |
| LMIC only | 29 (9.2) | 286 (90.8) |  |
| Other^c^ | 31 (9.2) | 304 (90.8) |  |
| **Country of corresponding author** |  |  | <0.0001 |
| Canada only or US only | 193 (17.7) | 900 (82.3) |  |
| EU only or UK only | 72 (14.0) | 443 (86.0) |  |
| LMIC only | 19 (9.9) | 173 (90.1) |  |
| Other^c^ | 11 (5.9) | 177 (94.1) |  |
| **Intervention type^d^** |  |  | <0.0001 |
| Clinical | 49 (8.4) | 531 (91.6) |  |
| Dietary and behavioral | 118 (13.1) | 782 (86.9) |  |
| Other | 128 (25.2) | 380 (74.8) |  |
| **Primary purpose^e^** |  |  | <0.0001 |
| Treatment | 58 (7.6) | 708 (92.4) |  |
| Health Services Research | 122 (34.2) | 235 (65.8) |  |
| Other | 115 (13.3) | 750 (86.7) |  |

^a^ Consists of 132 trials that did not report on consent, 139 that explicitly stated there was no consent, and 24 that explicitly stated that consent had been obtained for another aspect of the trial aside from study intervention.

^b^ Data for four trials were terminally missing and imputed the lowest journal impact factor of 0.11.

^c^ Other includes multinational trials.

^d^ Obtained from CT.gov and grouped as follows: Clinical (Drug, Device, Biological, Procedure, Radiation, Genetic, Combination Product, Diagnostic Test); Dietary Supplement and Behavioral; Other.

^e^ Categories obtained from CT.gov; Other includes Prevention, Diagnostic, Supportive Care, Screening, Basic Science, Educational/Counseling/Training, Other.

**Table S7: Sensitivity analysis showing bivariable tests of association with not obtaining consent, after excluding trials that did not include any statement about consent (N = 1856) (created by the authors)**

|  | **Obtained consent?** | |  |
| --- | --- | --- | --- |
|  | **No**  **(N = 163)** | **Yes**  **(N = 1693)** | **p-value** |
| **Publication year** |  |  | 0.044 |
| 2014-2015 | 48 (8.1) | 547 (91.9) |  |
| 2016-2017 | 51 (7.2) | 653 (92.8) |  |
| 2018-2019 | 64 (11.5) | 493 (88.5) |  |
| **Clinical trial start year** |  |  | <0.0001 |
| 1998-2010 | 31 (4.6) | 643 (95.4) |  |
| 2011-2012 | 49 (8.8) | 505 (91.2) |  |
| 2013-2018 | 83 (13.2) | 545 (86.8) |  |
| **Journal impact factor by tertile^a^** |  |  | 0.158 |
| Tertile 1: [0.11 – 3.575) | 49 (8.0) | 567 (92.0) |  |
| Tertile 2: [3.575 – 7.958) | 49 (8.1) | 554 (91.9) |  |
| Tertile 3: [7.958 – 70.67] | 65 (10.2) | 572 (89.8) |  |
| **Setting** |  |  | 0.097 |
| Public health | 26 (6.7) | 364 (93.3) |  |
| Clinical | 137 (9.4) | 1329 (90.6) |  |
| **Trial design** |  |  | <0.0001 |
| Individually randomised trial | 58 (4.6) | 1195 (95.4) |  |
| Cluster randomised trial | 105 (17.4) | 498 (82.6) |  |
| **Type of interventions in cluster trials (N = 688)** |  |  | <0.0001 |
| No professional-level or cluster-level interventions | 23 (8.7) | 241 (91.3) |  |
| Professional-level intervention, possibly other interventions, no cluster-level interventions | 15 (15.3) | 83 (84.7) |  |
| At least some cluster-level interventions | 67 (27.8) | 174 (72.2) |  |
| **Self-identified as pragmatic?** |  |  | <0.0001 |
| Yes | 69 (17.9) | 316 (82.1) |  |
| No | 94 (6.4) | 1377 (93.6) |  |
| **Country of study conduct** |  |  | <0.0001 |
| Canada only or US only | 102 (12.3) | 726 (87.7) |  |
| EU only or UK only | 29 (7.1) | 377 (92.9) |  |
| LMIC only | 12 (4.0) | 286 (96.0) |  |
| Other^b^ | 20 (6.2) | 304 (93.8) |  |
| **Country of corresponding author** |  |  | 0.0002 |
| Canada only or US only | 113 (11.2) | 900 (88.8) |  |
| EU only or UK only | 36 (7.5) | 443 (92.5) |  |
| LMIC only | 8 (4.4) | 173 (95.6) |  |
| Other^b^ | 6 (3.3) | 177 (96.7) |  |
| **Intervention type^c^** |  |  | <0.0001 |
| Clinical | 25 (4.5) | 531 (95.5) |  |
| Dietary and behavioral | 60 (7.1) | 782 (92.9) |  |
| Other | 78 (17.0) | 380 (83.0) |  |
| **Primary purpose^d^** |  |  | <0.0001 |
| Treatment | 33 (4.5) | 708 (95.5) |  |
| Health Services Research | 68 (22.4) | 235 (77.6) |  |
| Other | 62 (7.6) | 750 (92.4) |  |

^a^ Data for four trials were terminally missing and imputed the lowest journal impact factor of 0.11.

^b^ Other includes multinational trials.

^c^ Obtained from CT.gov and grouped as follows: Clinical (Drug, Device, Biological, Procedure, Radiation, Genetic, Combination Product, Diagnostic Test); Dietary Supplement and Behavioral; Other.

^d^ Categories obtained from CT.gov; Other includes Prevention, Diagnostic, Supportive Care, Screening, Basic Science, Educational/Counseling/Training, Other.

**REFERENCES**

1. Bioethics - CIOMS. https://cioms.ch/bioethics/ (accessed 2020 Oct 25).

2. Eldridge SM, Ashby D, Feder GS. Informed patient consent to participation in cluster randomized trials: an empirical exploration of trials in primary care. *Clin Trials* 2005;2(2):91–8 doi: 10.1191/1740774505cn070oa.

3. Anderson ML, Griffin J, Goldkind SF, et al. The Food and Drug Administration and pragmatic clinical trials of marketed medical products. *Clin Trials* 2015;12(5):511–9 doi: 10.1177/1740774515597700.

4. Integrated Addendum to ICH E6(R1): Guideline for Good Clinical Practice ICH E6(R2). https://database.ich.org/sites/default/files/E6_R2_Addendum.pdf 2016
